# PKH26 Staining Defines Distinct Subsets of Normal Human Colon Epithelial Cells at Different Maturation Stages

**DOI:** 10.1371/journal.pone.0043379

**Published:** 2012-08-22

**Authors:** Anna Pastò, Maddalena Marchesi, Adamo Diamantini, Chiara Frasson, Matteo Curtarello, Claudia Lago, Giorgia Pilotto, Anna Rosita Parenti, Giovanni Esposito, Marco Agostini, Donato Nitti, Alberto Amadori

**Affiliations:** 1 Department of Surgery, Oncology and Gastroenterology, University of Padova, Padova, Italy; 2 Hemato-Oncology Laboratory, Department of Pediatrics, University of Padova, Padova, Italy; 3 IRCCS Istituto Oncologico Veneto, Padova, Italy; 4 Department of Diagnostic Sciences and Special Therapies, University of Padova, Padova, Italy; National Cancer Institute, United States of America

## Abstract

**Background and Aim:**

Colon crypts are characterized by a hierarchy of cells distributed along the crypt axis. Aim of this paper was to develop an *in vitro* system for separation of epithelial cell subsets in different maturation stages from normal human colon.

**Methodology and Major Findings:**

Dissociated colonic epithelial cells were stained with PKH26, which allows identification of distinct populations based on their proliferation rate, and cultured *in vitro* in the absence of serum. The cytofluorimetric expression of CK20, Msi-1 and Lgr5 was studied. The mRNA levels of several stemness-associated genes were also compared in cultured cell populations and in three colon crypt populations isolated by microdissection. A PKH^pos^ population survived in culture and formed spheroids; this population included subsets with slow (PKH^high^) and rapid (PKH^low^) replicative rates. Molecular analysis revealed higher mRNA levels of both Msi-1 and Lgr-5 in PKH^high^ cells; by cytofluorimetric analysis, Msi-1^+^/Lgr5^+^ cells were only found within PKH^high^ cells, whereas Msi-1^+^/Lgr5^−^ cells were also observed in the PKH^low^ population. As judged by qRT-PCR analysis, the expression of several stemness-associated markers (Bmi-1, EphB2, EpCAM, ALDH1) was highly enriched in Msi-1^+^/Lgr5^+^ cells. While CK20 expression was mainly found in PKH^low^ and PKH^neg^ cells, a small PKH^high^ subset co-expressed both CK20 and Msi-1, but not Lgr5; cells with these properties also expressed Mucin, and could be identified *in vivo* in colon crypts. These results mirrored those found in cells isolated from different crypt portions by microdissection, and based on proliferation rates and marker expression they allowed to define several subsets at different maturation stages: PKH^high^/Lgr5^+^/Msi-1^+^/CK20^−^, PKH^high^/Lgr5^−^/Msi-1^+^/CK20^+^, PKH^low^/Lgr5^−^/Msi-1^+^/Ck20^−^, and PKH^low^/Lgr5^−^/Msi-1^−^/CK20^+^ cells.

**Conclusions:**

Our data show the possibility of deriving *in vitro*, without any selection strategy, several distinct cell subsets of human colon epithelial cells, which recapitulate the phenotypic and molecular profile of cells in a discrete crypt location.

## Introduction

Normal tissues are organized in a hierarchical fashion, where rare somatic cells endowed with stem-like properties give origin to a population of differentiated cells forming the bulk of tissue. The skin and the gastrointestinal tract undergo a tremendous turnover of the epithelial component, which entails the existence of a self-renewing population able to face the continuous replacement of the dying epithelial cells [1], [2]. These self-maintaining cells, called stem cells, are characterized by 4 fundamental properties: longevity, multipotency, quiescence, and asymmetric cell division. These two latter features, however, have been recently a matter of debate, as poor cell cycling and invariant asymmetric division may not represent obligatory properties of stem cells [3]–[6].

In the small intestine, the mucosa is organized into crypts and villi, populated by different types of cells that maintain their position along the intestinal architecture; in colon instead only crypts are found. While in the small intestine the location of stem cells is still debated [7]–[9], the situation is apparently less complicated at the colon level, where Paneth cells are not found [10], and stem cells may be identified at the very bottom of the crypt [11]. Surprisingly, despite the anatomy of the colon crypt being uniquely suited to study adult stem cells within their niche, only a few attempts have been made to isolate by laser microdissection the cells present in the different segments of the intestinal crypt, and to exploit their molecular profile as a gold standard [12].

Most information on the physiology of intestinal stem cells has been obtained in the mouse, as obvious limitations preclude transplantation or genetic marking approaches in humans. Very recently, Sato *et al.* [13] and Jung *et al.* [14] first succeeded in isolating and expanding *in vitro* normal human colonic stem cells by an experimental protocol which entailed the use of a complex combination of several growth factors [13] and a positive selection strategy based on the expression of the Ephrin type-B receptor 2 (EphB2) [14]. Here we derived *in vitro* spheroids from normal human colonic mucosa not by surface marker-driven selection, but by simply taking advantage of their slow proliferation rate in the absence of serum. In these spheroids, according to the phenotypic/molecular expression of several putative stemness markers including Leucine-rich repeat-containing G-protein coupled receptor 5 (Lgr5) [15], Musashi-1 (Msi-1) [16], B-lymphoma Mo-MLV insertion region 1 (Bmi-1) [17], EphB2 [18], Epithelial cell adhesion molecule (EpCAM) [19] and Aldehyde dehydrogenase 1 (ALDH1) [20], we could identify several discrete cell populations, in which mRNA expression profiles of specific genes closely mirrored the transcriptomic properties of epithelial cells isolated from different colon crypt portions by microdissection, as representative of different maturation stages.

## Materials and Methods

### Tissue Specimens and Cell Isolation

Following informed consent, 80 histologically normal and 5 tumoral human colonic mucosa samples were obtained from colon cancer-bearing patients undergoing colectomy. Immediately after resection, the tissues were washed in cold phosphate-buffered saline (PBS) containing Penicillin/Streptomycin, gentamicin (1 µl/ml) and amphotericin (1.25 µg/ml). Morphologically normal colon mucosa samples and tumor specimens were divided in two parts: one fragment was snap-frozen in liquid nitrogen, and stored at −80°C until use, while the other was processed as described elsewhere [21]. Briefly, the tissue was minced and incubated for 3 h at 37°C with collagenase (1.5 mg/ml) and hyaluronidase (20 µg/ml) in DMEM/F12 medium (Gibco, Invitrogen, Carlsbad, CA). The digested material was centrifuged and sequentially filtered through 70 and 40 µm filters; red blood cell lysis was performed at 37°C for 7 min in NH_4_Cl/KHCO_3_/EDTA buffer, and cell viability was assessed by Trypan Blue dye exclusion. The cell suspension was then plated in serum-free DMEM/F12 medium and maintained at 37°C in a 5% CO_2_ humidified atmosphere.

### *In vitro* Cell Culture and PKH26 Staining

Isolated cells from human colon mucosa specimens and cancer samples were plated at the concentration of 2×10^5^ cells/ml in untreated ultra-low adhesion 6-well plates (BD Falcon, Franklin Lakes, NJ) and cultured in serum-free DMEM/F12 medium supplemented with Pen/Strep, glucose (6 mg/ml), NaHCO_3_ (1 mg/ml), HEPES (5 mM), L-Glutamine (2 mM), heparin (4 µg/ml), bovine serum albumin (BSA; 4 mg/ml), insulin (25 µg/ml), anhydrous sodium selenite (30 nM), progesteron (20 nM), apo-transferrin (100 µg/ml), epidermal growth factor (EGF; 20 ng/ml), basic fibroblast growth factor (bFGF; 10 ng/ml), and putrescin (9.6 µg/ml).

After one week the cells were collected, washed with DMEM/F12 medium and incubated for 3 min with a 1∶250 (v/v) PKH26 solution (Sigma-Aldrich, St. Louis, MO) [22]. The staining was blocked with 1% BSA and DMEM/F12, and the cells were seeded in poly-2-hydroxyethyl methacrylate (PhEMA)-coated plates in the absence of serum at 2×10^4^ cells/well. According to the proliferation curve, the medium was replaced every 7 days. The plates were examined daily by phase-contrast microscopy to evaluate the formation of spheroid-like structures, and pictures were acquired with a Nikon camera. PKH26-stained cells were also observed with fluorescence microscope and pictures acquired with an Olympus camera or confocal microscope (Axiovert 1000M, Zeiss, Schwabhausen, Germany).

To address the frequency of spheroid-forming cells within normal colon epithelial cells, we performed an extreme limiting dilution analysis (ELDA) as previously described [23]–[24]. Briefly, at the 2^nd^ week of culture graded numbers of unfractionated and FACS-sorted PKH^pos^ cells were plated in 96-well flat-bottom ultra-low attachment PhEMA-coated plates (BD Falcon) in a total volume of 0.2 ml of medium as above. In all cases, at least 30 replicate wells were set up for each cell concentration. After 10 days of incubation at 37°C, 5% CO_2_, the wells were scored for outgrowth of spheroid-likes structures. The frequency of spheroid-forming precursors in each population was calculated using ELDA webtool at http://bioinf.wehi.edu.au/software/elda.

### Immunohistochemical Analysis

Immunohistochemical staining was performed on formalin-fixed, paraffin-embedded tissues using a standard avidin-biotin immunoperoxidase complex technique (Dako, Milan, Italy). Briefly, 4-µm thick sections of colon mucosa were mounted on silanized slides, dewaxed in xylene, dehydrated in ethanol, boiled in 0.01 M citrate buffer (pH 6.0) for 15 min in a microwave at 95°C, and incubated with 3% hydrogen peroxide for 5 min. After washing, the slides were incubated for 5 min in PBS containing 10% BSA, followed by incubation for 45 min at room temperature with the following mouse anti-human monoclonal antibodies (mAb): cytokeratin 20 (CK20, 1∶200; Abcam, London UK) or cytokeratin 18 (CK18, 1∶100; Dako). The incubation with rabbit anti-human Msi-1 (1∶100; Abcam) and mouse anti- human Lgr5 (1∶200; Origene, Rockville, MD), was performed overnight at 4°C. After washings, the sections were incubated with biotinylated goat anti-mouse immunoglobulin (LSAB kit, Dako) or anti-rabbit immunoglobulin (Vectastain ABC kit, Vector Lab, Burlingame, CA) and peroxidase-conjugated avidin (Dako). Lastly, 0.02% diaminobenzidine and 1% hydrogen peroxide (Dako) in PBS were used as substrates for the colorimetric reaction. Finally, the sections were counterstained with hematoxylin.

### Immunocytochemistry

PKH26-labeled cells obtained by dissociation of the spheroid structures and sorting (FACS ARIA II, BD Biosciences) were seeded onto poly-D-lysine-coated glasses at 5×10^4^ cells/spot, and fixed in 4% paraformaldehyde, followed by permeabilization with 0.1% Triton X-100 in PBS. Non-specific binding was prevented by saturation with PBS/5% BSA for 30 minutes. The cells were then incubated with the following anti-human mAb: Msi-1 (1∶100; Abcam), Lgr5 (1∶200; Origene), CK20 (Ks 20.8, 1∶10; Dako), CK18 (1∶200; Abcam), Mucin-1 (Muc-1, 1∶100; Abcam). For non-conjugated antibodies, secondary Alexa Fluor 488-conjugated goat anti-rabbit and Alexa Fluor 633-conjugated goat anti-mouse antibodies (1∶500; Molecular Probes, Invitrogen) were added for 30 minutes. Glasses were finally washed twice with PBS and mounted in glycerol. Pictures were acquired with confocal microscope.

### Immunofluorescence analysis on frozen sections

Immunofluorescence was performed on frozen human colon mucosa samples. Briefly, 7-µm thick sections were collected on Superfrost Plus microscope slides (Thermo Scientific, Munich, Germany) and dried at room temperature for 20 min. Sections were fixed in 4% paraformaldehyde and permeabilized with 0.1% Triton X-100. Non-specific binding was prevented by incubation in PBS 5% BSA for 1 h. Staining was carried out by incubating the sections with the anti-Msi-1 (1∶100; Abcam) and anti-CK20 (9∶50; Dako) antibodies for 30 minutes at room temperature. After three washes in PBS, appropriate secondary Alexa-conjugated antibodies (1∶500; Molecular Probes, Invitrogen) were added for 1 h. Nuclear staining was performed by propidium iodide incubation (1∶10.000; Invitrogen) and pictures acquired with confocal microscope at 20× and 40× magnification.

### Flow Cytometry (FACS) Analysis

Cell suspensions were collected weekly, washed in PBS, and PKH26 intensity measured by FACS (LSR II, BD Biosciences). The expression of CK18 (1∶200; Abcam), CK20 (1∶10; Dako), Lgr5 (1∶400; Origene), and Msi-1 (1∶200; Abcam) was measured by single staining or with the appropriate antibody combinations in fixed cells. After permeabilization with 0.1% Triton X-100 and blocking with PBS/5% BSA, the cells were incubated with the primary antibody and then stained with the appropriate secondary antibodies [25]. Data were collected from at least 5×10^4^ cells/sample and analyses were performed with Flow Jo (TreeStar, Ashland, OR). Cell viability was evaluated by Live/Dead (Invitrogen) staining.

### *In vitro* Cell Differentiation

To promote *in vitro* cell differentiation, cells maintained in serum free conditions were FACS-sorted at the 3^rd^ week of culture into PKH^pos^ and PKH^neg^ subsets, and included into Matrigel (BD Biosciences) or transferred to untreated 24-well plates, in the presence of 10% fetal calf serum (FCS, Invitrogen) and in the absence of bFGF and EGF. In both cases, the cells were plated at a density of 5×10^4^ cells/ml. For Matrigel cultures, 24-well plates were dispensed with Matrigel-included cells and layered with DMEM supplemented with 10% FCS. The cells were maintained at 37°C in a 5% CO_2_ humidified atmosphere, and the medium replaced every 2 days.

### Cells Isolation by Laser Microdissection

Snap-frozen colon biopsies were embedded in cryostat embedding medium (Bio Optica, Milan, Italy), cut into serial 7-µm sections and collected onto PEN-membrane slides (Leica, Wetzlar, Germany). Sections were fixed in 70% ethanol followed by nuclear staining with hematoxylin and dehydration in alcohol. Only longitudinally cut crypts were considered for microdissection. Three cell populations were isolated: putative stem-like cells at the very crypt base (2–3 cells/crypt), Transient-Amplifying (TA) cells located along the crypt wall, and terminally differentiated cells at the crypt apex and mucosal surface. Each population was isolated by a laser microdissection system (Leica DM6000 B), and collected on the test tube cap. For each population at least 1×10^4^ cells were collected in the appropriate amount of TRIzol (Invitrogen), and kept at −80°C until RNA extraction.

### RNA Extraction, Reverse Transcription and Quantitative Real-Time PCR (qRT-PCR) Assay

Total RNA was extracted from FACS-sorted populations, spheroids, *in vitro* differentiated cells, and microdissected samples by the TRIzol method according to the manufacturer's instructions. cDNA was synthesized from 0.5–1 µg of total RNA using the Superscript II reverse transcriptase (Invitrogen). Fifty-five ng of cDNA were used as a template and mixed with 10 µl of 2× Platinum SYBR Green qPCR SuperMix-UDG (Invitrogen) and primers (Table 1) in a reaction volume of 20 µl. Cycling conditions were 10 min at 95°C, 60 cycles of 15 s at 95°C and 1 min at 60°C. Each sample was run in duplicate on ABI PRISM® 7900HT Sequence Detection System (PE Biosystems, Foster City, CA). Results were analyzed as described [26] using the comparative ΔΔCt method: data are presented as the fold difference in gene expression normalized to the housekeeping gene β_2_-microglobulin and relative to a relevant reference sample. qRT-PCR efficiency was always in the range 95–105%.

**Table 1:** Sequences of the primers used in qRT-PCR experiments.

Gene name	Forward	Reverse
Lgr5	5′-CTCTTCCTCAAACCGTCTGC-3′	5′-GATCGGAGGCTAAGCAACTG-3′
Msi-1	5′-GGTTTCCAAGCCACAACCTA-3′	5′-TCGGGGAACTGGTAGGTGTA-3′
CK20	5′-GACGCCAGAACAACGAATACC-3′	5′-ACGACCTTGCCATCCACTAC-3′
β2Micro	5′-TCTCTCTTTCTGGCCTGGAG-3′	5′- TCTCTGCTGGATGACGTGAG-3′
Bmi-1	5′-GCATCGAACAACGAGAATCA-3′	5′-TACCCTCGACAAAGCACACA-3′
EpCAM	5′-CCAGAACAATGATGGGCTTT-3′	5′-GCACTGCTTGGCCTTAAAGA-3′
ALDH1	5′-CCCGTTGGTTATGCTCATTT-3′	5′-TGCTCTGCTGGTTTGACAAC-3′
Muc-1	5′-AGTTCAGGCCAGGATCTGTG-3′	5′-GAAATGGCACATCACTCACG-3′
Muc-2	5-′ACCCGCACTATGTCACCTTC-3′	5′-GGGATCGCAGTGGTAGTTGT-3′
EpHB2	5′-TCCATCTGGGACTTTCAAGG-3′	5′-TGTTGATGGGACAGTGGGTA-3′

### Western blotting

After 3 weeks of culture, FACS-sorted PKH^pos^ cells and *in vitro* differentiated cells were harvested, lysed and subjected to SDS-polyacrylamide gel electrophoresis and Western blotting (WB). The membrane was saturated with PBS 5% non-fat dry milk (Sigma-Aldrich) for 1 h at room temperature. Immunoreactivity was evaluated by hybridization using the following antibodies: rabbit anti-Msi-1 (Abcam, 1∶2000 final dilution), mouse mAb anti-Muc-1 (Abcam, 1∶1000 final dilution), and mouse mAb anti-α tubulin (Sigma Aldrich, 1∶5000 final dilution). The blots were then hybridized with a 1∶5000 dilution of HRP-conjugated anti-mouse or anti-rabbit antibody (Amersham-Pharmacia, Little Chalfont, U.K.). Finally, the signal was detected by chemiluminescence with SuperSignal kit (Pierce, Rockford, IL).

### Statistical Analysis

Data of replicate experiments were shown as mean value ±1 Standard Deviation (SD). Comparisons between groups were made by the Student's t-test.

## Results

### Identification of a Slowly Cycling Cell Population and *In Vitro* Spheroid Formation

**Figure 1:**
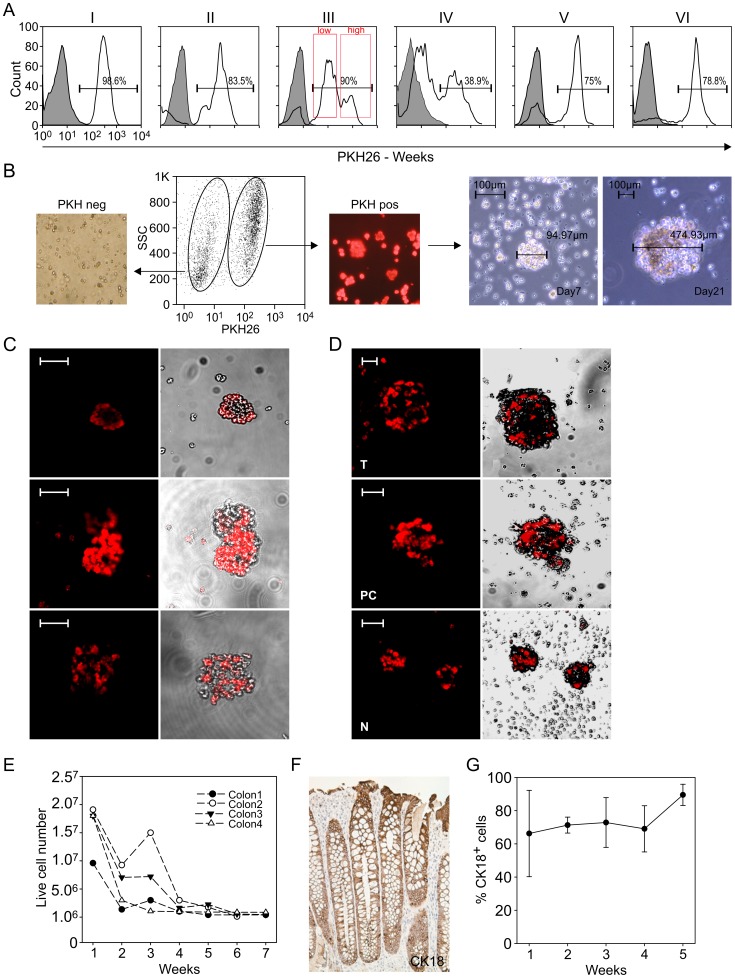
Kinetics of PKH26 and In vitro Spheroid Formation. (A) Cells obtained from human normal colon samples were cultured *in vitro* as detailed in *Materials and Methods*, and stained with PKH26. Cytofluorimetric analysis of PKH staining over 6 weeks of culture showed the progressive selection of a cell population expressing the dye at high intensity. At the 3^rd^ week of culture, within the PKH^pos^ subset it was possible to distinguish two major peaks (PKH^high^ and PKH^low^). One representative experiment is shown. (B) Three weeks after PKH26 staining, PKH^pos^ and PKH^neg^ cells were FACS-sorted and cultured under serum-free conditions as detailed in *Materials and Methods*. While PKH^neg^ cells rapidly died in culture (left panel), PKH^pos^ cells began to form spheroid-like structures, which progressively grew over time in culture (right panels; magnification 20×, scale bars: 100 µm). (C) Confocal microscopy analysis of spheroids showed cells with different intensity of PKH26 staining at 3 weeks of culture; magnification 20×. (D) Spheroids obtained after 3-week culture from PKH^pos^ cells isolated from tumor [T] and pre-cancerous [PC] human colon samples were morphologically comparable to those obtained from normal [N] colon tissue. Scale bars: 100 µm. (E) Kinetics of cell survival in culture; in the absence of serum, the number of living cells decreased until about 5% of input cells at the end of the culture period. Results obtained in 4 consecutive experiments are shown. (F) CK18 staining of a human colon mucosa section (10× magnification). (G) Kinetics of CK18 expression in cultured colon cells; data are expressed as mean values ± SD of six different experiments.

Following isolation from fresh normal colon mucosa samples, the cells were stained *in vitro* with PKH26, a dye which binds membrane phospholipids, thus conferring a bright red fluorescence [22]. PKH26 staining may represent an unconventional proliferative assay and a surrogate marker of stemness, akin DNA labels such as Bromodeoxyuridine. In fact, the dye segregates during each cell division, and a progressive decrease of fluorescence intensity in daughter cells can be observed [27], allowing to monitor by FACS the cells which proliferate more slowly based on their brighter appearance. Immediately after staining, >98% of the cells were PKH^pos^ (Fig. 1A); in the subsequent weeks, a progressive increase in the number of cells expressing the dye at low intensity (PKH^low^) was observed, until virtually only cells with high PKH26 expression (PKH^high^) remained (Fig. 1A), indicating that the cultures contained cells with different replicative potential.

Several studies have demonstrated that under specific culture conditions one of the major properties of cancer stem cells is their ability to form spheroids *in vitro* [28]–[30]. When PKH^pos^ and PKH^neg^ cells were FACS-sorted at the 3^rd^ week after PKH staining, and cultured in the absence of serum as detailed in *Materials and Methods*, within a few days PKH^pos^ cells began to form small, round cell clusters which grew into spheroid structures (Fig. 1B), whereas sorted PKH^neg^ cells died within 2–6 days, and no spheroid-like structures could be observed. Confocal microscopy analysis revealed that these spheroids contained a heterogeneous mixture of cells with different PKH26 staining intensity and cells negative for the dye, often spatially segregated within a same structure (Fig. 1C). By a limiting dilution assay, in three consecutive experiments the frequency of spheroid-forming cells was ranged for 1/100 to 1/220 PKH^pos^ cells, whereas, as expected, this frequency was much lower when unfractionated epithelial cells were plated (Table 2). Microscopic evaluation showed that spheroids obtained from purified PKH^pos^ cells from normal colon mucosa were fully comparable to those obtained from PKH^pos^ cells isolated by the same technique from colon tumors and pre-cancerous lesions after 3 weeks of culture (Fig. 1D).

**Table 2:** Extreme limiting dilution analysis of spheroid-forming cell precursors in normal colon epithelial cell populations.

Number of cells/well	Number of wells plated	Number of wells showing spheroids	
		Unfractionated cells	PKH^+^ cells
10,000	30	30	30
2,000	30	18	30
400	30	9	26
80	30	2	16
16	30	0	12
3	30	0	5
Spheoids forming frequency (95% CI)		1/1,748 (1/1,217–1/2,509)	1/107 (1/75–1/152)

P-value<0.0001.

Data were pooled from three indipendent experiments. CI, confidence interval.

On the other hand, the surviving PKH^pos^ subset was a minority of the original population, as the total number of cells in culture decreased over time, and by 6 weeks only about 5% of the cells originally present at the beginning of the culture could be recovered (Fig. 1E). Flow cytometry analysis for CK18, a known marker of colon epithelium (Fig. 1F), revealed that early in culture the majority of the cells also expressed CK18 (Fig. 1G); this percentage increased over time, with >95% of the surviving cells positive for both PKH26 and CK18 by 5 weeks of culture (Fig. 1G). Altogether, the above results pointed to the existence within epithelial colon cells of a slowly dividing subset able to maintain PKH26 staining *in vitro*, which could be a suitable candidate for a stem cell-containing population.

### Stemness Marker Expression by *in vitro* Cultured Colon Cells

**Figure 2:**
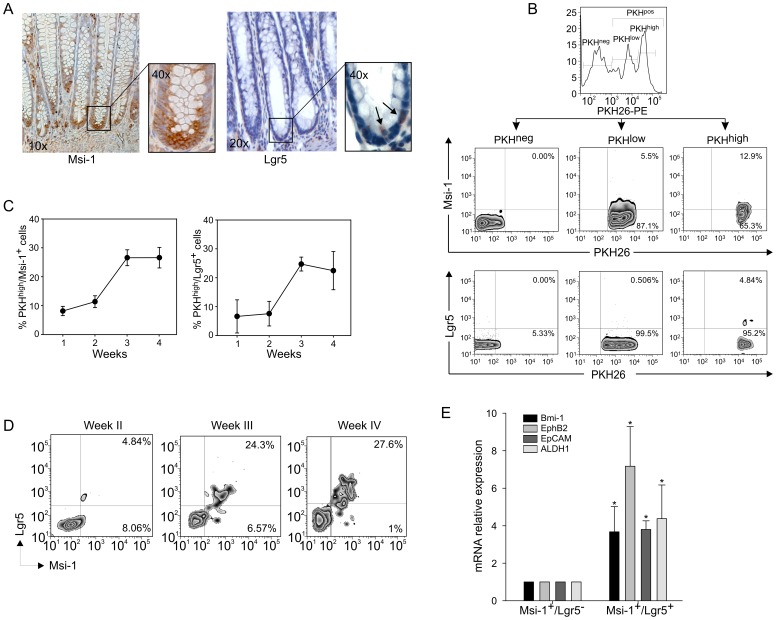
Expression of Stemness-Associated Markers in Cultured Colon Cells. (A) Immunohistochemical analysis of Msi-1 and Lgr5 expression in human normal colon biopsies; Msi-1^+^ and Lgr5^+^ cells are localized at the crypt base, where *bona fide* stem-like cells home; magnification is indicated in the boxes. (B) Cytofluorimetric analysis of Msi-1 and Lgr5 expression by PKH26-labelled cells expressing different intensities of PKH26 staining (PKH^neg^, PKH^low^ and PKH^high^; upper panel) at the 3^rd^ week of culture. Msi-1^+^ cells were found within both PKH^high^ and PKH^low^ cell populations (middle panels), whereas Lgr5^+^ cells were confined to the PKH^high^ subset (lower panels). One representative experiment out of three consecutive is shown. (C) Cytofluorimetric analysis of Msi-1 and Lgr5 expression by PKH^high^ cells over 4 weeks of culture. Data are expressed as percent mean values (± SD) of 5 consecutive experiments. (D) Kinetics of Msi-1 and Lgr5 expression by PKH^high^ cells at the 2^nd^, 3^rd^ and 4^th^ week of culture. All Lgr5^+^ cells also co-expressed Msi-1, whereas a small fraction of Msi-1^+^ cells did not express Lgr5. One representative experiment out of 4 is shown. (E) qRT-PCR analysis of expression of Bmi-1, EphB2, EpCAM and ALDH1 in FACS-isolated Msi-1^+^/Lgr5^−^ and Msi-1^+^/Lgr5^+^ cell subsets at the 3^rd^ week of culture. Data were calculated as mean values ± SD as detailed in *Materials and Methods*, normalized to the housekeeping gene β_2_-microglobulin and relative to the reference sample (Msi-1^+^/Lgr5^−^ cells). * p<0.05.

To characterize the cell population responsible for spheroid generation, we analyzed the expression of two putative markers of colon stem-like cells, Msi-1 [16] and Lgr5 [15], [31]. As judged by immunohistochemistry on colon biopsies, Msi-1 staining was substantially confined to the crypt basement (Fig. 2A, left panel), where colon stem cells are expected to reside [32], while the paucity of Lgr5-stained cells at the very crypt bottom (Fig. 2A, right panel) fully confirmed previous data [33]. By three-color cytofluorimetry at the 3^rd^ week of culture, Msi-1^+^ cells were mainly confined within the slowly cycling PKH^high^ cell population, but a sizable fraction of PKH^low^ cells also expressed this marker, whereas no Msi-1 expression could be detected in PKH^neg^ cells (Fig. 2B, middle panel). Lgr5 expression, instead, was entirely restricted to PKH^high^ cells, and no Lgr5 expression was recorded in epithelial cells showing lower PKH26 levels (Fig. 2B, lower panel).

Flow cytometry analysis revealed that the percentage of Msi-1^+^ and Lgr5^+^ cells increased over the culture period (Fig. 2C); at the 4^th^ week of culture 28.7±2.4% of the PKH^high^ cells expressed the Msi-1 antigen, while 24.9±3.6% expressed Lgr5, thus indicating an enrichment of Msi-1^+^ and Lgr5^+^ cells in the PKH^high^ population. As shown in Figure 2D, at all times of culture Lgr5^+^ cells invariably co-expressed Msi-1, and this subset consistently augmented over the culture period, while a small population of Msi-1^+^ cells that did not express Lgr5 was found, which underwent progressive reduction late in culture (Fig. 2D).

We also compared in FACS-purified Msi-1^+^/Lgr5^−^ and Msi-1^+^/Lgr5^+^ cells the expression of other putative markers for colon stem cells, such as Bmi-1 [17], EphB2 [18], EpCAM [19], and ALDH1 [20]. qRT-PCR analysis of FACS-sorted cells after 3 weeks of culture revealed that the mRNA expression levels of all these genes were consistently higher (p<0.05) in Msi-1^+^/Lgr5^+^ than in Msi-1^+^/Lgr5^−^ cells (Fig. 2E). Thus, these findings seemed to indicate that the Msi-1^+^/Lgr5^+^ cell population is strongly enriched in stem-like cells, compared to Msi-1^+^/Lgr5^−^ cells. Altogether, the above data seemed also to delineate the existence of a sort of stemness “gradient” in the cultured population, where at least 3 discrete cell subsets could be identified, a PKH^high^/Lgr5^+^/Msi-1^+^ population (reasonably reflecting relatively quiescent cells endowed with most prominent stem-like properties), a PKH^high^/Lgr5^−^/Msi-1^+^ subset (likely reflecting cells with a slow division rate, which lost the expression of a key stemness marker such as Lgr5), and a PKH^low^/Lgr5^−^/Msi-1^+^ population which had likely undergone more cycles of replication.

### Stemness and Differentiation Marker Co-Expression by *in vitro* Cultured Cells

**Figure 3:**
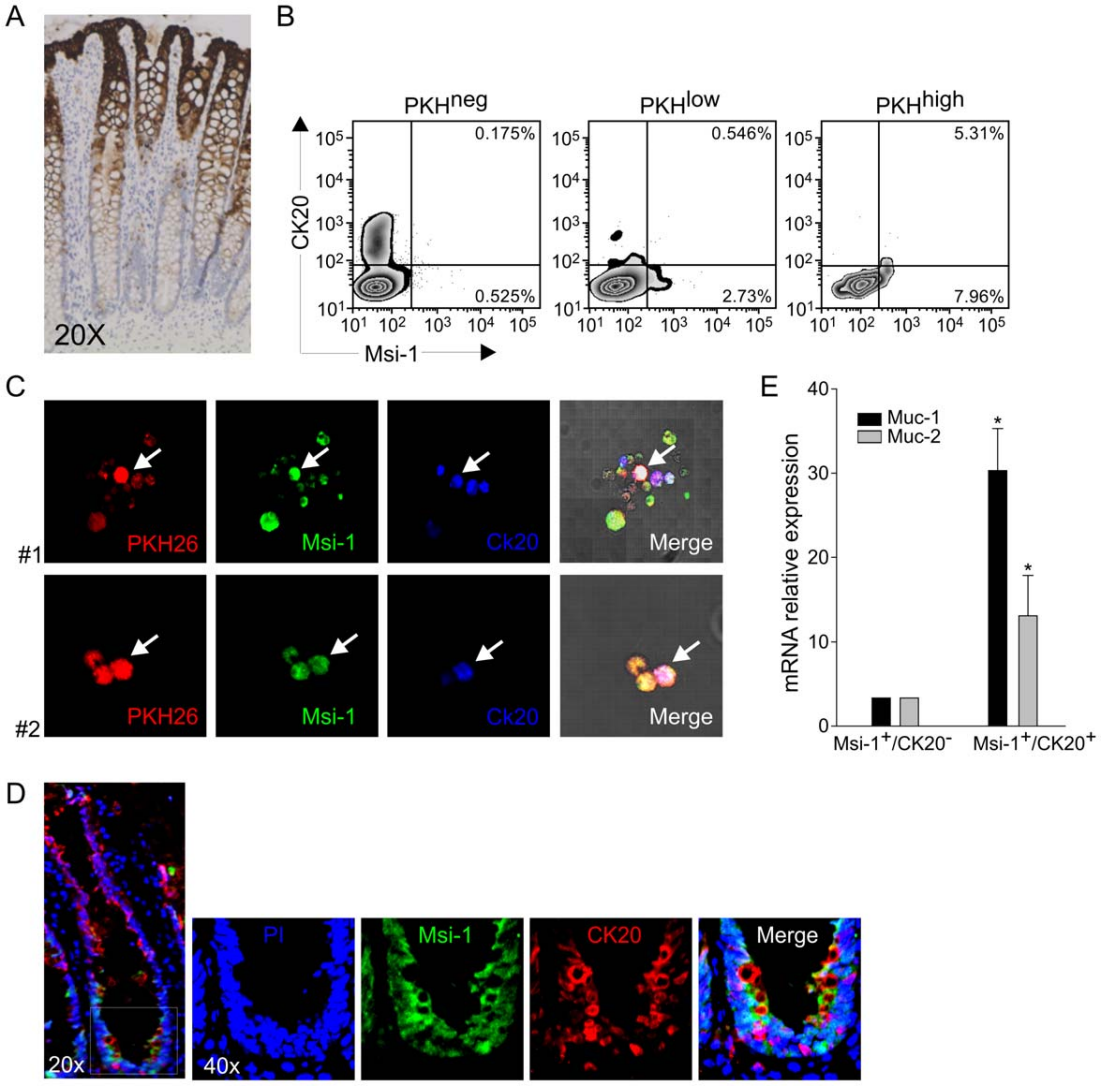
Expression of Msi-1 and CK20 in Cultured Colon Cells. (A) Immunohistochemical analysis of CK20 showed labeling of the epithelial cells along the crypt wall and at the mucosal surface, but not at the crypt bottom. (B) Cytofluorimetric analysis of Msi-1 and CK20 expression by PKH^neg^, PKH^low^ and PKH^high^ populations at the 3^rd^ week of culture; a small subset of PKH^high^ cells co-expressed both markers. One representative experiment out of 4 consecutive is shown. (C) Confocal microscopy analysis of CK20 and Msi-1 expression in PKH^pos^ cells. The cells co-expressing both markers are indicated by an arrow. Magnification 40×. (D) Immunofluorescence analysis of Msi-1 and CK20 expression in frozen colon sections. Double-positive cells are evident at the very crypt base. (E) qRT-PCR analysis of Muc-1 and Muc-2 expression on FACS-sorted Msi-1^+^/CK20^−^ and Msi-1^+^/CK20^+^ subsets at the 3^rd^ week of culture. Data are calculated as mean values ± SD as detailed in *Materials and Methods*, normalized to the housekeeping gene β_2_-microglobulin and relative to the reference sample (Msi-1^+^/CK20^−^ cells). * p<0.05.

CK20 is considered as a marker of differentiated intestinal epithelial cells [34]; a preliminary confirmation of the ability of anti-CK20 antibody to identify differentiated epithelial cells was obtained by immunohistochemistry in colon sections (Fig. 3A). CK20 expression was mostly confined, as expected, to PKH^neg^ cells (Fig. 3B), which reasonably underwent more cycles of replication. Surprisingly, although the vast majority of CK20^+^ cells did not express Msi-1 (Fig. 3B), double staining with anti-Msi-1 and anti-CK20 antibodies of cells cultured for 3 weeks revealed the presence of a small population expressing both markers only in the PKH^high^ subset (Fig. 3B). In 4 consecutive experiments, Msi-1^+^/CK20^+^ cells accounted for 5.13±0.32% of this most brilliant population. No co-expression of Lgr5 and CK20 was observed (not shown), and the Msi-1^+^/CK20^+^ population was virtually absent in the PKH^low^ subset. The presence of a small population expressing both Msi-1 and CK20 was also supported by confocal microscopy analysis of cells cultured for 3 weeks (Fig. 3C). In view of the possible artefactual nature of these findings, we addressed the presence of these double-positive cells in colon crypt sections. As shown in Figure 3D, the *in vivo* presence of Msi-1^+^/CK20^+^ cells could be demonstrated by immunofluorescence analysis of frozen colon sections. This observation confirms previous data by Dalerba *et al.* [35], who identified CK20^+^ goblet cells in close proximity to the more immature cellular compartment of the crypt bottom. Indeed, quantitative PCR analysis of FACS-sorted Msi-1^+^/CK20^+^ and Msi-1^+^/CK20^−^ cells at the 3^rd^ week of culture revealed a significantly higher expression of Muc-1/2 in the double-positive subset (Fig. 3E).

### Molecular Analysis of *in vitro* Cultured Cells and Microdissected Colon Crypt Populations

**Figure 4:**
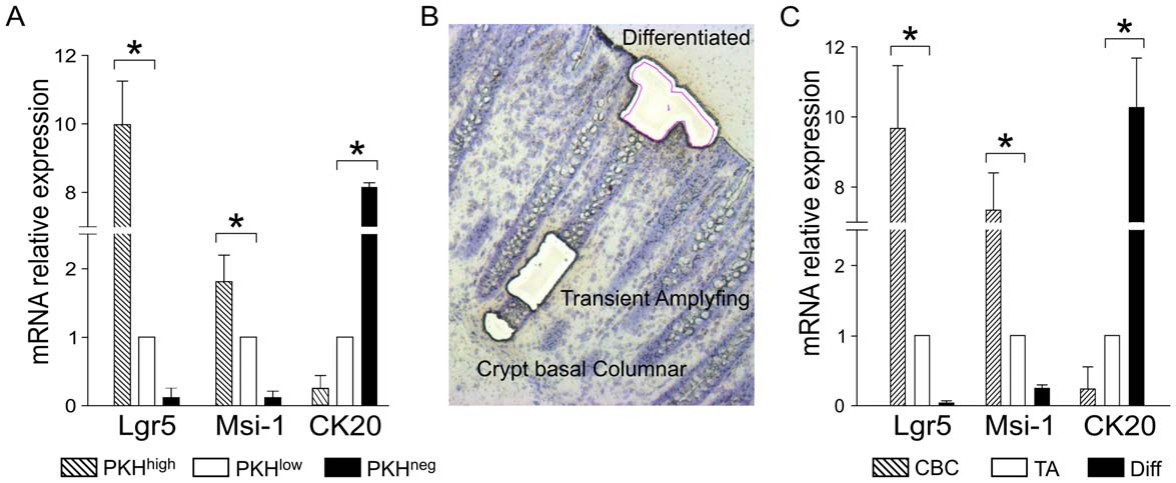
Comparison by qRT-PCR of Stemness and Differentiation Marker Expression in Cultured Colon Cells and Microdissected Colon Crypt Populations. mRNA levels of Lgr5, Msi-1 and CK20 genes were evaluated by qRT-PCR on FACS-sorted PKH^high^, PKH^low^ and PKH^neg^ cells at the 3^rd^ week of culture (Panel A) and on Differentiated (Diff), TA and CBC cells (Panel C) isolated by laser microdissection from colon crypts (as shown by the picture reported in Panel B). Data are shown as mean values ± SD calculated as described in detail in *Materials and Methods*, normalized to the housekeeping gene β_2_-microglobulin and relative to the reference sample (PKH^low^ for sorted cultured cells, and TA for cells isolated by microdissection). * p<0.05.

The expression of genes putatively reflecting different stemness and differentiation stages was also compared by qRT-PCR in PKH^high^, PKH^low^ and PKH^neg^ cells sorted after 3 weeks of culture, and in cells isolated from different colon crypt portions by microdissection. PKH^high^ cells showed significantly higher (p<0.05) mRNA levels of both Msi-1 and Lgr-5, as compared to PKH^low^ cells, while no expression of these genes was detected in the PKH^neg^ subset (Fig. 4A). As expected, opposite results were obtained for CK20 mRNA levels, which were significantly higher in the PKH^neg^ and PKH^low^ cell subset (Fig. 4A). By taking advantage of the discrete location of the cells within the colon mucosa, the above results were compared to mRNA levels of the same genes in three colon cell populations isolated through laser microdissection (Fig. 4B): cells at the very crypt bottom (which *bona fide* include Crypt Basal Columnar, CBC, cells and would correspond to cultured PKH^high^ cells), TA cells along the crypt wall (which are reasonably representative of cultured PKH^low^ cells) and terminally differentiated cells at the crypt apex and mucosal surface (likely reflecting PKH^neg^ cells). As expected, Msi-1 and Lgr-5 mRNA levels were much higher in the basal crypt cell population, and barely or not detectable at all in terminally differentiated cells (Fig. 4C). Thus, these data further buttressed the idea that the *in vitro* derived PKH^high^ population could indeed contain colon cells endowed with stem-like properties,

### *In Vitro* Differentiation of Cultured Colon Cells

**Figure 5:**
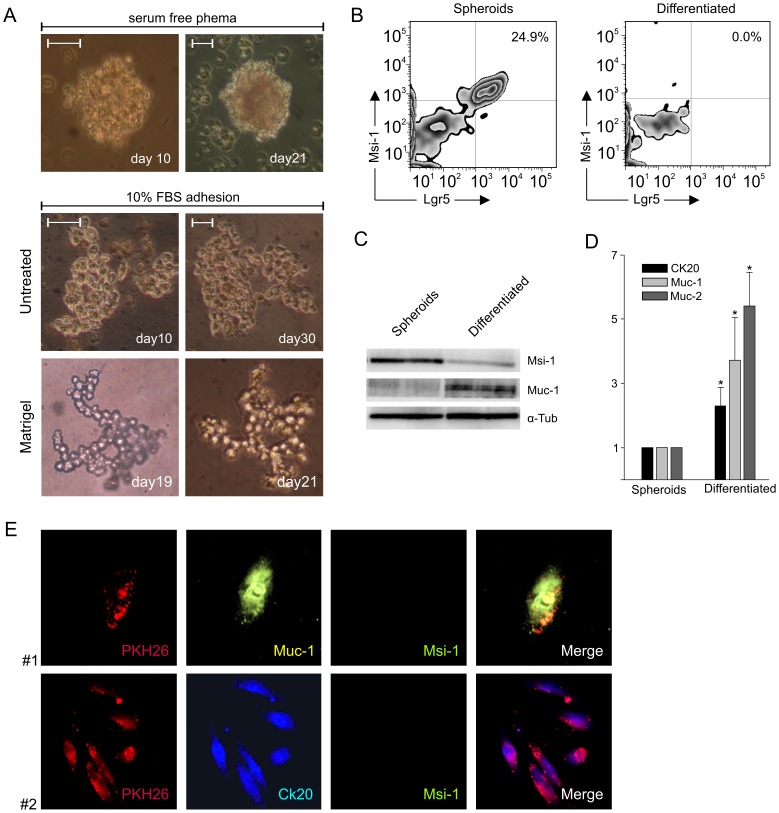
*In vitro* Differentiation of Cultured PKH^pos^ Cells. (A) After 3 weeks of culture the spheroids obtained from PKH-stained cells maintained in serum-free medium in non-adherent plates (upper panel) were dissociated, and PKH^pos^ cells were FACS-sorted and cultured in the presence of 10% FCS in adherent plates or included into Matrigel. In both conditions, the cells changed their morphology (lower panel), and in the presence of Matrigel they formed branched structures surrounding a “hole”. (B) Cytofluorimetric analysis of Msi-1 and Lgr5 expression in PKH^pos^ cells cultured for 3 weeks in serum-free conditions (Spheroids) and after 10 days in differentiating conditions (Differentiated). (C) WB analysis of Msi-1 and Muc-1 proteins in Spheroids and Differentiated cell lysates. α-tubulin was used as a control for protein contents. (D) qRT-PCR analysis of CK20, Muc-1 and Muc-2 expression in Spheroids and Differentiated cells. Data are calculated as mean values ± SD as detailed in *Materials and Methods*, normalized to the housekeeping gene β_2_-microglobulin and relative to the reference sample (spheroid cells). *p<0.05. (E) Confocal microscopy analysis of differentiated cells. In the presence of serum and adhesion conditions, the cells presented a fragmented PKH26 dye pattern along the cellular membrane, completely loosing the expression of Msi-1 while acquiring Muc-1 (sample # 1) and CK20 (samples #2) expression.

To evaluate the differentiation potential of PKH26-stained cells, PKH^pos^ and PKH^neg^ subsets were sorted at the 3^rd^ week of culture, and cultured in the presence of 10% FCS, in both uncoated and Matrigel-coated tissue culture plates. Under both culture conditions, PKH^neg^ cells died within 10 days (not shown), whereas PKH^pos^ cells survived but did not form spheroids, as they did in the absence of serum (Fig. 5A, upper panel). On the other hand, under differentiating conditions these cells showed a substantial proliferative potential and the ability to grow into organized structures; as shown by phase-contrast microscopy, in the presence of serum PKH^pos^ cells adhered to the plate assuming an epithelial morphology (Fig. 5A, middle panel), while in the presence of Matrigel, although not forming organoids similar to those recently described by Sato et al. [13], they generated branched structures of cells growing around “holes” (Fig. 5A, lower panel). Cytofluorimetric analysis revealed that the differentiated cells completely lost Msi-1 and Lgr5 expression just after 10 days of culture in serum and adhesion conditions (Fig. 5B). These data were also confirmed by WB analysis (Fig. 5C), which showed significantly higher levels of Msi-1 protein in spheroids, compared to differentiated cells; these latter showed instead much higher levels of Muc-1 protein than cells grown under non-differentiating conditions (Fig. 5C). Furthermore, by comparing RNA extracted from both PKH^pos^ spheroid-forming cells and differentiated cells, we demonstrated an enrichment in the expression of the differentiation markers CK20, Muc-1 and Muc-2 (p<0.05) in PKH^pos^ cells maintained in adhesion condition and in the presence of serum for 10 days (Fig. 5D). Immunofluorescence analysis showed a partial loss of the PKH26 membrane labeling, which assumed a patchy pattern at the cells surface (Fig. 5E); furthermore, these cells expressed the differentiation markers Muc-1 (sample #1) and CK20 (sample #2), whereas Msi-1 expression was no longer detectable (Fig. 5E).

## Discussion

Aim of this paper was to address the *in vitro* culture and characterization of distinct epithelial cell subsets from normal human colon mucosa. In general, previous attempts to grow in long-term culture cells with stem-like properties from normal human colon mucosa had been unsuccessful [36]. Very recently, however, Sato *et al.* [13] and Jung *et al.* [14] succeeded in isolating and expanding *in vitro* normal human colonic stem cells by a positive selection strategy targeting the expression of EphB2. In this paper, we instead adopted a totally different strategy, and chose to derive cells endowed with stem-like properties by simply exploiting their proliferation rate. After dissociation from the biopsy, the cells were labelled with a membrane dye (PKH26) which was progressively “diluted” at the cell surface proportionally to the replication rate, and let to grow in serum-free medium.

Our study demonstrated that the ability to form spheroids in serum-free medium is not restricted to tumor and pre-cancerous samples [28]–[30], but that epithelial cells isolated from normal colon biopsies and maintained in stem cell medium are also able to form spheroidal structures, morphologically comparable to those obtained from tumor colon samples. This ability was confined to a slowly cycling PKH^pos^ population, which survived *in vitro* in the absence of serum for up to 40 days. Indeed, after 6 weeks of culture the surviving population accounted for less than 5% of the input population; these cells, however, maintained a bright PKH26 staining, and were of epithelial origin, as virtually all the cells expressed a pan-colon marker such as CK18. When transferred to a serum-containing medium, these cells underwent differentiation, as judged by the acquisition of adhesive properties, a different morphology, the loss of stemness markers, and the expression of typical differentiation markers such as CK20 and Muc-1/2.

The major achievement of our work is the *in vitro* culture and characterization of several discrete cell populations, some of which endowed with more prominent stem-like properties. In this regard, we focused our attention on two commonly recognized markers of colon stem cells, such as Msi-1 and Lgr5. Msi-1 is a RNA-binding protein that plays an essential role in regulating asymmetric cell division, by maintaining colon stem/progenitor cell proliferation and inhibiting the differentiation process [37]; Lgr5 is supposed to be a Wnt target gene, and its expression is lost as a consequence of the inhibition of the Wnt pathway [11]. Msi-1 and Lgr5 expression was evaluated in PKH^high^, PKH^low^ and PKH^neg^ cell subsets, as representative of cell populations which underwent increasing cycles of replication. Cytofluorimetric analysis showed that Lgr5 expression was strictly confined to the PKH^high^ subset, and all Lgr5^+^ cells also expressed Msi-1. On the contrary, even though Msi-1 was mainly expressed by PKH^high^ cells, not all Msi-1^+^ cells also expressed Lgr5, and a small proportion of Msi-1^+^ cells could also be found within PKH^low^ cells. In addition, while no co-expression of CK20 and Lgr5 could be documented, we identified a small subset of Msi-1^+^ cells which also expressed the differentiation marker CK20. Thus, based on PKH26 staining and stemness/differentiation marker expression, several distinct populations could be distinguished in our *in vitro* cultures: I) a PKH^high^/Lgr5^+^/Msi-1^+^/CK20^−^ subset, which likely reflects a relatively quiescent stem-like population that did undergo minimal *in vitro* proliferation; II) a PKH^high^/Lgr5^−^/Msi-1^+^/CK20^+^ population that did undergo poor proliferation but expressed a differentiation marker; III) a PKH^low^/Lgr5^−^/Msi-1^+^/CK20^−^ subset, where part of the cells which underwent active replication still maintained the expression of a stemness marker; IV) a PKH^low^/Lgr5^−^/Msi-1^−^/CK20^+^ subset; and V) a PKH^neg^/Lgr5^−^/Msi-1^−^ subset.

The location of the two small “transitional” subsets (PKH^high^/Lgr5^−^/Msi-1^+^/CK20^+^ cells and PKH^low^/Lgr5^−^/Msi-1^+^/CK20^−^ cells) within the developmental tree of colon epithelial cells is unclear. While both had lost the expression of one of the mostly recognized stemness markers, the former likely underwent poor replication (in view of its high PKH26 staining) and continued to express Msi-1 but in combination with a differentiation marker; the second subset (PKH^low^/Lgr5^−^/Msi-1^+^/CK20^−^) instead, while continuing to express some properties of immature cells, underwent proliferation without acquiring a differentiated phenotype. To our mind, the apparently contrasting properties of PKH^high^/Lgr5^−^/Msi-1^+^/CK20^+^ cells, which also express markers typical of goblet cells (Fig. 3), could reflect a sort of “committed” subset [36], where the relatively immature cells quickly acquire the expression of classical differentiation markers (such as CK20 and Muc-1/2), without showing a significant proliferative potential, as demonstrated by the maintenance of high levels of PKH26 staining. On the other hand, PKH^low^/Lgr5^−^/Msi-1^+^/CK20^−^ cells might represent the natural evolution of a rapidly cycling immature reservoir, which did not acquire differentiation markers but also did not completely loose self-maintaining properties. Within the limits of an analysis carried out in cells maintained *in vitro* in experimental, non-physiologic conditions, our observations might reconcile the debate on the relative quiescence of stem-like cells in the gastrointestinal tract [3], [6], by showing that cells with differential expression of commonly recognized stemness markers may coexist and show different replicative potential. In any case, even though we demonstrated the possibility of dissecting *in vitro* discrete cell subsets with distinct stemness/differentiation profiles, we are well aware that the maturation and differentiation process of colon epithelial cells is a continuous pathway which in humans may be difficult or even impossible to “freeze” in discrete steps. Indeed, recent work [8] clearly demonstrated in mice an interconversion between intestinal stem cell populations endowed with an active/quiescent status according to their location in distinct niches; in addition, alternative stem cell pools may coexist, in which Bmi-1 expressing cells may vicariate the absence of Lgr5^+^ cells in a complex hierarchical model [9].

Our phenotypic data are buttressed by the analysis of the transcriptomic profile of the above stemness/differentiation markers in PKH^neg^, PKH^low^ and PKH^high^ cells, compared to that observed in cells obtained by the microdissection of distinct crypt portions (Fig. 4B). The cells isolated at the very bottom of the crypt expressed huge amounts of mRNA for both Msi-1 and Lgr5; on the other hand, differentiated cells at the very top of the crypts only showed high levels of CK20, and no Msi-1/Lgr5 mRNA was detected. While these *ex vivo* results were not surprising, it is noteworthy that they fully mirrored the figures observed in the corresponding subsets expanded in culture (Fig. 4), further buttressing the notion that PKH^high^ cells do contain a cell population endowed with stem-like properties. The exclusive presence of Lgr5^+^ cells within the subset maintaining high PKH26 expression and the absence of Lgr5/CK20 co-expression strongly indicates a prominent role of Lgr5 as a stem cell marker, confirming previous data in mice [38]. In addition, the finding that other commonly recognized stemness markers (such as Bmi-1, EphB2, EpCAM and ALDH1) are mostly expressed in Lgr5^+^/Msi-1^+^ cells, also strongly points to Lgr5 as a very marker of stemness, compared to Msi-1.

In conclusion, our data showed the possibility of deriving *in vitro*, without any selection strategy or driving pressure, an array of cellular subsets differentially expressing the most common stemness and differentiation markers of human colon epithelial cells, which recapitulate the phenotypic and molecular profile of cells in an anatomical position compatible with stem-like cells. By this approach, it was also possible to identify cells with intermediate maturational patterns, which share markers reminiscent of a stem stage and markers reflecting the transition to more mature stages, and that can be identified *in vivo* in the array of cells populating the crypt bottom. Further work will allow exploring in the different subsets of *in vitro* cultured cells mRNA/microRNA expression profiles, as well as their epigenetic features, to better characterize the discrete stages of progression towards maturity of human colon stem cells [39]–[40]. Indeed, this could constitute a solid background to further understand the complex and still elusive biology of their malignant counterpart, and help clarifying the possible role of cancer stem cells as election targets for anti-tumor therapy.
